# Sociodemographic characteristics of persons committing suicide in Durban, South Africa: 2006–2007

**DOI:** 10.4102/phcfm.v6i1.568

**Published:** 2014-02-24

**Authors:** Soornarain S. Naidoo, Lourens Schlebusch

**Affiliations:** 1 Department of Family Medicine, University of KwaZulu-Natal, South Africa; 2 Department of Behavioural Medicine, University of KwaZulu-Natal, South Africa

## Abstract

**Background:**

Suicidal behaviour is a leading contributor to the burden of disease worldwide and varies widely between countries. South African figures are amongst the highest in the world, with recent trends indicating a disturbing rise, especially amongst the younger age groups, across all races.

**Aim:**

This study analysed sociodemographic characteristics and trends relating to suicides committed in Durban, South Africa during the period of 2006–2007.

**Method:**

A retrospective analysis of suicidal deaths (during 2006–2007), extracted from autopsy registers at all three government-run mortuaries in Durban, was conducted.

**Results:**

The total number of suicides in Durban increased by 6.68% from 2006 to 2007. Suicide accounted for an average of 8.8% of all non-natural deaths per year of the study. The overall suicide rates of 14.53 (2006) and 15.53 (2007) per 100 000 population are comparable with national and global figures. The majority of suicides occurred in single unemployed persons, men and younger age groups. The largest number of suicides per year was recorded in black people, followed by Indian, white and mixed-race people. Hanging was the preferred method in the majority of victims, followed by self-poisoning, shooting and jumping.

**Conclusions:**

The findings indicate a disturbingly high suicide rate amongst the various population and age groups in Durban. The dominant methods used may be influenced by ease of access. The reported trends may worsen unless there is a swift and decisive public health response and cohesive community-based programmes which include a supportive multidisciplinary network.

## Abstrait

**Caractéristiques sociodémographiques des personnes qui se sont suicidées à Durban, en Afrique du Sud: 2006–2007**

**Présentation:**

Le comportement suicidaire est l'un des principaux facteurs contribuant à la charge mondiale de morbidité et varie dans une large mesure d'un pays à l'autre. L'Afrique du Sud enregistre l'un des taux de suicide les plus élevés du monde, les tendances récentes révélant une hausse dérangeante, notamment chez les plus jeunes, toutes races confondues.

**Objectif:**

Cette étude a consisté en l'analyse des caractéristiques sociodémographiques et des tendances associées aux suicides enregistrés à Durban, en Afrique du Sud, au cours de la période 2006-2007.

**Méthode:**

Une analyse rétrospective des décès par suicide (en 2006-2007), recensés par le biais des rapports d'autopsie dans les trois morgues publiques de Durban, a été réalisée.

**Résultats:**

Le nombre total de suicides à Durban a augmenté de 6.68 % entre 2006 et 2007. Le suicide était en moyenne la cause de 8.8 % des décès de causes non naturelle au cours de l'année concernée par l’étude. Les taux de suicide généraux de 14.53 (2006) et 15.53 (2007) pour 100 000 personnes sont comparables avec les chiffres enregistrés dans le pays et dans le monde. Les personnes qui s’étaient suicidées étaient célibataires et sans emploi, de sexe masculin et appartenaient à la tranche d’âge des plus jeunes. Le plus grand nombre de suicides par an a été enregistré chez des individus d'origine africaine, suivi par des individus d'origine indienne, européenne et métisse. La mort par pendaison était la méthode privilégiée par la majorité des victimes, suivie par l'ingestion de substances toxiques, les armes à feu et les sauts.

**Conclusions:**

Les conclusions révèlent un taux de suicide particulièrement élevé chez les jeunes des différents groupes de population à Durban. Les principales méthodes utilisées pourraient être justifiées par leur facilité d'accès. Les tendances révélées par ce rapport pourraient s'aggraver en l'absence d'intervention de santé publique rapide et décisive et de programme communautaire cohésif accompagné d'un réseau d'entraide pluridisciplinaire.

## Introduction

Suicidal behaviour (comprising suicidal ideation, planning, attempts and completed or fatal episodes) has increased globally over the last decade and has been identified as being one of the major contributors to the high disease and healthcare burden in many low- to middle-income countries.^1^ According to the World Health Organization (WHO), approximately 1.53 million people will die annually from suicide by 2020, compared with the 0.88 million suicides that occurred in 2002.^2^ Suicide rates are higher amongst men (with male to female ratios up to 3:1) throughout the world except in China, where female rates are consistently higher than those for men, particularly in rural areas.^3, 4^ Globally, the majority of suicides occur in people aged 35–44 years in both sexes and the ratio of suicide attempts to suicide varies from 20–40:1.^2^

In South Africa, it was reported in 2008 that approximately 6500 suicides and 130 000 suicide attempts occurred annually.^5^ It is widely believed that these figures have since increased.^1^ Non-fatal suicidal behaviour in South Africa outnumbers suicides, with the ratio varying between 8:1 and 20:1, depending on the geographical area and the racial group of the study sample.^6^ The average age for suicide nationally is 34 years and the highest number of suicides occur in the 15–19 year age group, followed by the 10–14 year group.^7^ Suicide rates have been increasing steadily in all population groups in the country over the last decade, although the prevalence varies considerably across age and race groups. Suicide has been labelled as the third major cause of death in Indian, black and mixed-race people after homicide and natural deaths and it is the second major cause of deaths in white people.^8, 9, 10^

A wide range of methods is used by persons committing suicide.^1^ These depend on a number of factors such as the mental state of the suicidal person; intention to die (high or low); intensity of the trigger factor or stressor and the ensuing crisis; threshold or tolerance to trigger factors; personal or popular choices in terms of knowledge of prior effectiveness of the method; access to the agent or instrument to be used; and the environment where the act is planned to take place. At a global level, shooting, hanging and self-poisoning have remained the leading agents of choice.^4, 6, 11, 12^ Figures released for South Africa in the last decade have shown that hanging and shooting were the most preferred methods, followed by other methods such as self-poisoning with agents such as pesticides and poisons, drug overdoses, self-gassing and self-immolation.^13, 14, 15, 16, 17^ An analysis of the autopsy register in the Eastern Cape Province in South Africa (previously called Transkei) for the period 1993–2003 showed an increasing trend of hangings from 5.2 per 100 000 in 1993 to 16.2 per 100 000 in 2003, particularly in the younger (20–29 year) age group.^13^ This is consistent with other studies on choice of method in South Africa.^6^

Homicide-suicide and extended suicide, especially involving adult men, intimate partners, and/or family members, have increased notably in South Africa, attracting much publicity, mainly because of their associated physical trauma and psychosocial impact. Several case study reports have been published highlighting characteristics of the perpetrator (usually male and often employed in the security or police services, or unemployed); the victim (usually female); the location of the event (usually the home); and the method generally used (firearms).^6, 18, 19, 20^

Durban is the third-largest city in South Africa and the largest in KwaZulu-Natal Province. It accounts for 1.4% of the geographical area of the KwaZulu-Natal Province but, according to the 2001 national population census, was home to 3.2 million (34%) of the province's population.^21^ Sixty percent of the province's economic activity takes place in this port city, with 68% of its inhabitants being of a working age (15–65 years) and 27.9% being unemployed, 88.6% of whom are black South Africans. A demographic analysis of the city in 2001 showed that 68.3% of the residents were black, 2.9% mixed-race, 19.9% Indian and 8.9% white, with 51.94% being women and 48.06% men. The reported age profile of the city's inhabitants in 2001 is reflected in Table 1.^21^

**TABLE 1:** Durban population age groupings.

Age	0–14	15–24	25–44	45–64	65 +
Residents (%)	27.7	21.2	32.5	14.5	4.2

*Source*: Statistics South Africa^21^

This study was part of a larger study carried out by the lead researcher during the period 2007–2011,^1^ and was intended to analyse the existing suicide trends amongst the different population groups in Durban, South Africa, as well as sociodemographic characteristics of persons who committed suicide in Durban during the period 2006–2007.

## Research method and design

Medico-legal autopsies of all unnatural deaths occurring in the Durban municipality are routinely carried out in three state mortuaries (Phoenix, Gale Street and Pinetown). Final cause of death is entered into the autopsy record following the routine inquest procedure. This process, including finalisation of the inquest docket prepared for each case and authentication of the exact cause of death, can sometimes take as long as two years.

This epidemiological study was conducted in 2010–2011. A retrospective analysis of the autopsy registers at the three government-run mortuaries in the Durban Municipality was conducted for the period 2006–2007. All suicidal deaths were further extracted from these registers and analysed. The rationale for choosing the above study period was to ensure that only authentic suicide cases finalised via the inquest methodology employed in South Africa were included for analysis in the study. Cases that were classified as ‘deaths due to other causes’ or those that could not be finalised via the inquest process were excluded from this study.

### Ethical considerations

Ethical approval to conduct the study was granted by the University of KwaZulu-Natal's Biomedical Research Ethics Committee (Reference number HSS/0181/06D) and the KwaZulu-Natal Provincial Department of Health.

### Data analysis

All data were captured and analysed using the Statistical Software Package for Social Sciences^®^ (SPSS) version 19 (IBM Corp, Armonk, NY, 2010) and SAS/STAT software version 8.2 (Cary, NC, SAS Institute, 2010).

## Results

### Prevalence of suicide in Durban

During the two-year study period, 6046 and 5550 non-natural deaths were recorded for 2006 and 2007 respectively. Suicide accounted for 7.7% (*n* = 465) of these non-natural deaths (*N* = 6046) in 2006 and 9.0% (*n* = 497) of non-natural deaths (*N* = 5550) in 2007. The suicide incidence rate for Durban in 2006 was 14.53/100 000 population and that for 2007 was 15.53/100 000 population. These calculations were based on the 2001 national census figures.^21^

### Suicide victim characteristics

Male suicides outnumbered female suicides (Table 2) in both 2006 (ratio 3.2: 1) and 2007 (3.6: 1).This was also reflected in the monthly mean calculations for both years. The monthly mean for male suicides was 27.54 (SD = 10.2) in 2006 and 32.17 (SD = 7.3) in 2007, whereas that for females was 9.33 (SD = 3.8) in 2006 and 8.83 (SD = 2.4) in 2007. In both years, the largest number of suicides was recorded in black people, followed by Indian, white and mixed-race people (Table 2).

**TABLE 2:** Suicide frequencies by sex and race per year.

Suicide frequency variables	2006		2007	
	
	*n*	%	*n*	%
Sex				
Male	356	76.2	387	78.3
Female	111	23.8	107	21.7
Race				
Black	267	57.2	289	58.4
Mixed race	10	2.1	15	3.0
Indian	124	26.6	136	27.5
White	66	14.1	55	11.1

*Source*: Data collated from mortuary records

### Monthly frequencies for all groups

**FIGURE 1:**
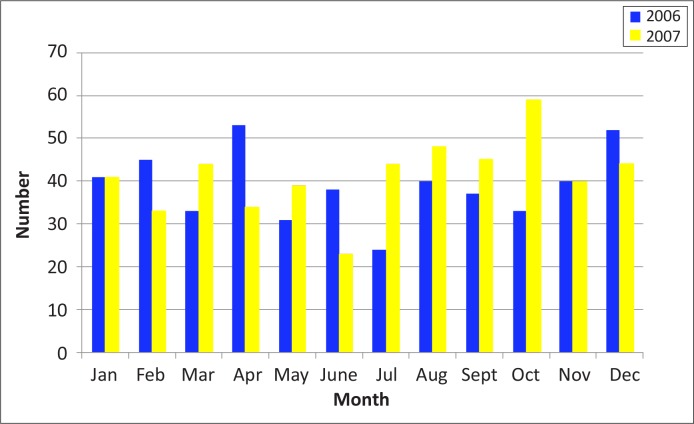
Suicide frequency by month and year. *Source*: Data collated from mortuary records

The mean number of suicides per month in 2006 was 36.0 (SD = 12.9) and that in 2007 was 41.1 (SD = 8.8). The monthly frequency peaked beyond 50 per month twice in 2006 (April and December) and once in October 2007 (Figure 1).

Monthly frequencies expressed as means for each race group within Durban per year also indicated a larger number for black people compared with the other race groups in both 2006 and 2007 (Table 3). The suicide rate per 100 000 had increased from 2006 to 2007 for all race groups except for white people (Table 3).

**TABLE 3:** Suicide frequencies by race, monthly mean and rates per year of study.

Race group	2006		2007	
	
	Monthly Mean (SD)	Suicide Rate/100 000	Monthly Mean (SD)	Suicide Rate/100 000
Black	22.4 (6.0)	12	23.9 (6.4)	13
Mixed race	1.4 (0.8)	11	1.9 (1.1)	17
Indian	10.3 (4.2)	19	11.3 (3.8)	21
White	5.1 (3.0)	23	4.6 (2.3)	19

*Source*: Data collated from mortuary records

SD, standard deviation.

**FIGURE 2:**
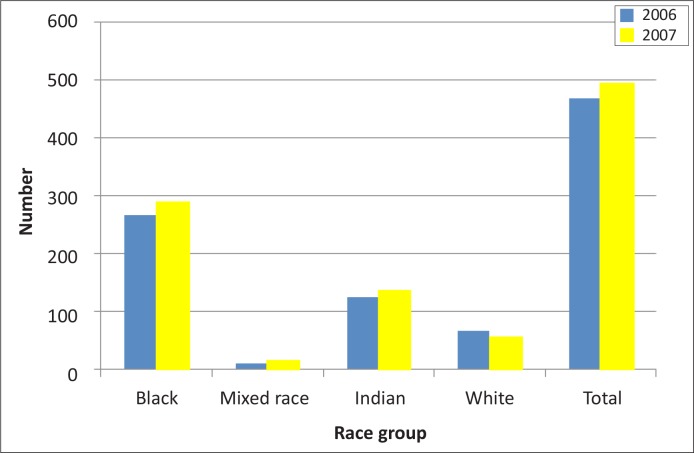
Number of suicides by race. *Source*: Data collated from mortuary records

In terms of race and gender (Table 4 and Figure 2), the majority of suicides in both years were in black men and women, with a rise in the number of black men and a shift reduction in the number of black women between 2006 and 2007. There was a small increase in both mixed-race and Indian men and women, a decrease in white men and a small increase in white women.

**TABLE 4:** Number of suicides by race and gender for 2006 and 2007.

Gender and year	Black	Mixed race	Indian	White	Gender Total	Gender%	Year total
Men 2006	202	7	91	53	353	75.9	465
Women 2006	63	3	32	14	112	24.1	
Men 2007	239	10	103	40	392	78.9	497
Women 2007	51	5	34	15	105	21.1%	
Total	**555**	**25**	**260**	**122**	**962**	**-**	**-**
% of Total	**58.0%**	**2.0%**	**27.0%**	**13.0%**	**100.0%**		

*Source*: Data collated from mortuary records

**FIGURE 3:**
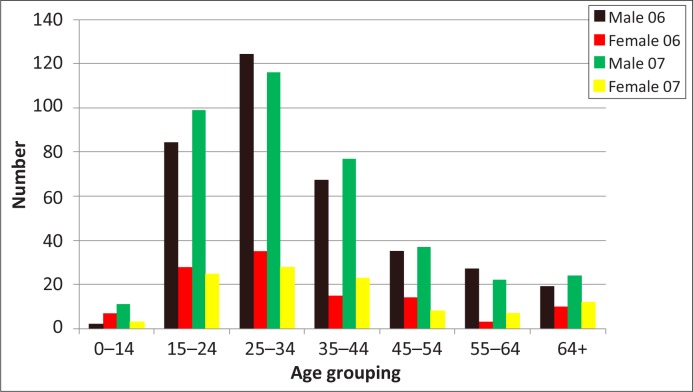
Suicide victims by gender and age. *Source*: Data collated from mortuary records

The aggregated data indicates that most of the suicides occurred in people between the ages of 15 and 44 years (Figure 3). The majority of suicides took place amongst the 25–34 year olds, followed by the 15–24 and the 35–44 year age groups. In the under-14 age group, there were 13 boys (two in 2006 and 11 in 2007) and 10 girls (seven in 2006 and three in 2007). Most of the male age groups experienced an increase from 2006 to 2007 except in the 25–34 and 55–64 year olds. There was a decrease in the number of women between 2006 and 2007 in most categories except in the 35–44 and 55 years and older groups.

The overall mean age (95% CI) at which people committed suicide was 34.14 years (95% CI 32.80–35.48) in 2006 and 34.63 (95% CI 33.29–35.97) in 2007. The lowest age had dropped from 9 years in 2006 to 5 years in 2007, as had the highest age from 98 to 95 (Table 5).

**TABLE 5:** Age descriptors of suicide victims by year.

Age	2006	2007
Mean age (95% CI) in years	34.1 (32.8–35.5)	34.6 (33.3–36.0)
Median age (SD) in years	30.0 (14.6)	31.0 (5.1)
Age range in years	9–98	5–95

*Source*: Data collated from mortuary records

SD, standard deviation; CI, confidence interval.

Of those for whom marital status was recorded, 60.6% in 2006 and 58.3% in 2007 were single (Table 6). Married persons accounted for 23.4% and 22.0% in 2006 and 2007 respectively.

**TABLE 6:** Marital status of suicide victims by year of study.

Marital status	2006		2007	
	
	*n*	%	*n*	%
Single	282	60.6	290	58.3
Married	109	23.4	107	22.0
Separated	0	0	1	0.2
Divorced	15	3.2	18	3.4
Widowed	3	0.6	10	2.0
Not Recorded	55	11.8	71	14.1

**Total**	465	100%	497	100%

*Source*: Data collated from mortuary records

In both 2006 and 2007, the majority of people who committed suicide were unemployed (43.4% in 2006 and 38.5% in 2007), followed by general workers (12.1% in 2006 and 14.1% in 2007), other varied occupations (11.2% in 2006 and 9.5% in 2007) and students (7.5% in 2006 and 5.6% in 2007).These figures are not shown in the Tables.

### Breakdown of preferred methods of suicide

**FIGURE 4:**
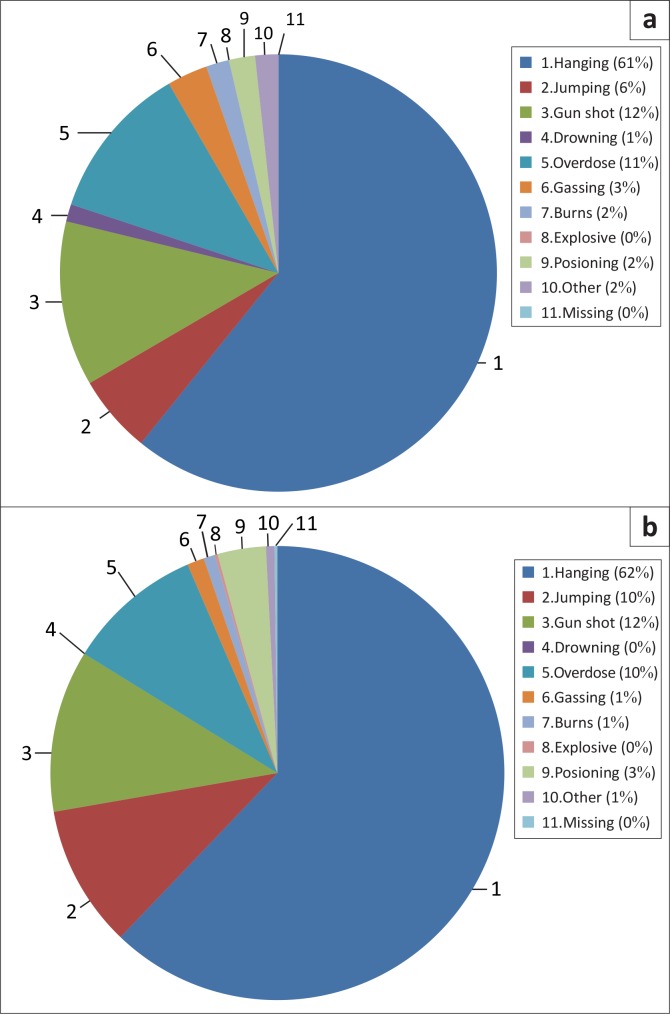
Most common suicide methods in (a)2006 and (b) 2007. *Source*: Data collated from mortuary records

Overall, of the nine main methods used to commit suicide (Figure 4), hanging accounted for nearly two-thirds in both 2006 (61%) and 2007 (62%).This was followed by guns, jumping from heights and overdose. These four methods accounted for 88% of all suicides in 2006 and 94% of all suicides in 2007.

**FIGURE 5:**
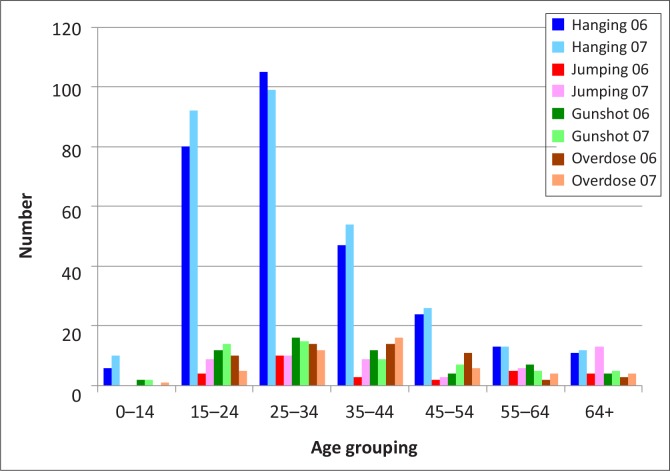
Age groupings and the four most common methods of suicide. *Source*: Data collated from mortuary records

Hanging was the preferred method of suicide for all age groups except for those over 65 years in 2007, where jumping from high-rise buildings was the preferred method (Figure 5). In both the 15–24 and 25–34 year age groups, gun usage was the next most common method used, whilst in the 35–44 and 45–55 year age groups, overdose was the method of choice, followed by hanging and gun usage.

## Discussion

This study was part of a larger study undertaken by the researcher during the period 2006–2011, some of the findings of which have been used in partial fulfilment of a doctoral degree.^1^ The salient findings of this study in particular support and complement those found by Donson and others in 2007.^22^ The total number of suicides confirmed in this study (*n* = 495) for Durban during 2007 was greater than that shown in the Donson study (*n* = 398).^22^ The difference may be explained by the differing methodologies used in each study. There are no other recent relevant studies.

This study showed a gradient of increase in suicides over the two-year period studied, which is consistent with the reported gradual increase in suicide rates for the country as a whole from around 8% in 1999 to 11% currently.^6, 23^ Compared with suicide rates for the other major South African cities, the rates per 100 000 population in this study shows that Durban has a high suicide rate, which is also comparable with the average global rate.^22^

Although the results of the present study indicate that suicides occur throughout the year in Durban, there were discernible peaks at particular times, namely, early and toward the end of the year, which is consistent with other findings for the country.^6, 23^ It has been postulated that this finding may be linked to high degrees of stress toward the year end, which have been precipitated by socio-economic difficulties and academic expectations, particularly amongst our youth.^6^

This study also demonstrated that the actual number of suicides per year of study varied considerably between racial groupings in Durban – it was twice as high for black people as for Indian people, the former constituting the largest racial or population group in the metropolitan area. Figures for mixed-race people and white people were much lower. These findings are in sharp contrast with earlier studies on suicide rates for the different race groups in South Africa, which showed that white South Africans consistently outnumbered black, Indian and mixed-race South Africans.^8, 9, 24^ The suicide gradient amongst the different race groups per year showed an increase from 2006 to 2007 in all groups except for white South Africans.

The fact that the majority of suicides in both years were committed by men is consistent with those found in other studies.^9, 15, 25, 26, 27, 28^ At least two-thirds of the suicide victims in our research were unmarried, widowed or divorced. Similar findings have been reported in other studies with some reporting that separated men were at higher risk of committing suicide compared with separated women.^29, 30^

In terms of age groupings, the majority of suicides in our study took place amongst 25–34 year olds, followed by 15–24 year olds and 35–44 year olds. Other local studies have reported comparable findings,^25, 31^ which is similar to the global trend of a shift to younger people committing suicide.^2^ However, a study in Dar es Salaam, Tanzania, showed that the 45–59 year group had the highest suicide rate,^27^ although varying figures are reported for Africa.^32^ The high number of suicides committed by young students in the present study is a source of great concern and is consistent with previous South African research findings. Research findings have linked this trend to a variety of pre-disposing and risk factors. These include child abuse and neglect, interpersonal problems, peer pressure, substance abuse, family dysfunction and unrealistic academic expectations.^6, 33, 34, 35^

The majority of suicide victims in our study were unemployed or casual workers. It is possible that socio-economic issues played a role in influencing a fatal suicidal outcome in these victims. This is supported by other studies which have shown an inverse relationship between income and psychological distress.^36^ Also, a number of researchers have offered various related explanations regarding the deteriorating trend in suicides in South Africa. These include the inability to cope with new roles since the abolition of apartheid and the introduction of a democracy since 1994; acculturation and western lifestyle influences and moving away from protective traditional lifestyles; socio-economic pressures, with resulting interpersonal and family problems; inappropriate family and peer expectations; increasing urbanisation; increasing competitiveness; and rising unemployment in a changing South African society since democracy.^6, 37, 38^

The methods used in committing suicide depend on availability, knowledge of lethality and access to the method by the victim. In our study, hanging emerged as the dominant method in at least two-thirds of suicide victims, all racial groups, both genders and all ages, except in the over-65 year age group in 2007 where jumping was the chosen method. This was followed by other methods such as guns, jumping from heights and over-dosing of medicines. In general, hanging was the preferred method amongst unemployed victims (65.5%) compared with those who were employed at the time (56.6%). Interestingly, in the Eastern Cape Province of South Africa, hanging was reported to be the preferred method in uneducated (70%) and unemployed (64%) victims, whilst the more highly educated (17%) used firearms to commit suicide.^39^ The prominence of suicide by hanging has also been documented in a number of other studies^13, 40^ and differs from those in a recent study conducted in Malawi where self-poisoning emerged as the being the most common method used by both men and women.^41^

## Limitations

The data recorded in this study may not be truly representative for several reasons. Only data relating to confirmed suicides were extracted from mortuary registers and analysed in this study. It is possible that these registers may have contained administrative errors, omissions and inaccurate recordings, meaning that the number of suicides analysed may thus have been underestimated. The study also relied on the accuracy of the data entries recorded in mortuary registers in Durban. Some of the data may have been obtained from contacts other than next-of-kin and may not have been accurate. Missing data were noted in particular categories studied, such as marital status, occupation and method of suicide.

All suicides committed in Durban during the study period were analysed. This may have included persons not normally resident in Durban, therefore the figures presented in this study may not be truly representative of the residents domiciled in Durban.

The study design unfortunately precluded an exploration of precipitating factors and causes. Relatives and family members could not be interviewed. In instances where suicide notes were left by victims, details within these notes were filed in inquest files and not made part of the mortuary registers.

## Conclusion

The actual rates of suicide per racial group showed a gradient increase from 2006 to 2007 for all groups except for white people. Health and education policy makers, counsellors and community leaders should respond to the disturbingly-high suicide figures recorded amongst certain categories of Durban residents such as black and Indian people, the unemployed and the youth, including scholars. This study endorses the call made by many researchers and policy makers for the enactment of a national policy framework in South Africa that would embrace a comprehensive multidisciplinary primary care approach to the current burden of suicidal behaviour in the country. Hanging emerged as the dominant method used by the majority of those committing suicide mainly because of its simplicity, proven effectiveness and easy access to structures within or adjacent to homes. There is therefore an urgent need for innovative employment strategies to help the unemployed, as well as greater awareness and education campaigns (amongst public health policy makers, low-cost housing developments and families) designed to restrict such easy access to any potential method by vulnerable individuals.

A larger and more extensive study over a longer period is recommended in order to confirm the reported trends.

## References

[1] Naidoo SS. The impact of a brief intervention programme on suicidal behaviour in a South African community [unpublished Doctoral dissertation]. Durban: Department of Family Medicine, University of KwaZulu-Natal; 2012.

[2] Bertolote JM, Fleischmann A. Suicidal behavior prevention: WHO perspectives on research. Am J Med Genet C Semin Med Genet. 2005;133C(1):8–12. http://dx.doi.org/10.1002/ajmg.c.30041, PMid:15645530 15645530 10.1002/ajmg.c.30041

[3] Phillips MR, Li X, Zhang Y. Suicide rates in China,1995–1999. Lancet 2002;359(9309):835–840. http://dx.doi.org/10.1016/S0140-6736(02)07954-0 11897283 10.1016/S0140-6736(02)07954-0

[4] Kong Y, Zhang J. Access to farming pesticides and risk for suicide in Chinese rural young people. Psychiatry Res. 2010;179(2):217–221. http://dx.doi.org/10.1016/j.psychres.2009.12.005, PMid:, PMCid: 20483175 10.1016/j.psychres.2009.12.005 PMC2925062

[5] Burrows S, Schlebusch L. Priorities and prevention possibilities for reducing suicidal behaviour In: A Van Niekerk, S Suffla, M Seedat, editors. Crime, violence and injury prevention in South Africa:data to action. Tygerberg: MRC-UNISA Crime,Violence and Injury Lead Programme, 2008; p. 173–201.

[6] Schlebusch L. Suicidal behaviour in South Africa. Pietermaritzburg: University of KwaZulu-Natal Press; 2005 PMCid:

[7] Matzopoulos R, Cassim M, Seedat M. A profile of fatal injuries in South Africa: Fourth Annual Report of the National Injury Mortality Surveillance System. Pretoria: Medical Research Council; 2003.

[8] Burrows S, Vaez M, Butchart A, et al The share of suicide in injury deaths in the South African context: sociodemographic distribution. Public Health. 2003;117(1):3–10. http://dx.doi.org/10.1016/S0033-3506(02)00019-7 12802898 10.1016/s0033-3506(02)00019-7

[9] Scribante L, Blumenthal R, Saayman G, et al. A retrospective review of 1018 suicide cases from the capital city of South Africa for the period 1997-2000. Am J Forensic Med Pathol. 2004;25(1):52–55. http://dx.doi.org/10.1097/01.paf.0000113862.03302.1d, PMid: 15075690 10.1097/01.paf.0000113862.03302.1d

[10] Prinsloo M. A profile of fatal injuries in South Africa. Cape Town: South African Medical Research Council; 2002.

[11] Asirdizer M, Yavuz MS, Aydin SD, et al. Suicides in Turkey between 1996 and 2005: general perspective. Am J Forensic Med Pathol. 2010;31(2):138–145. http://dx.doi.org/10.1097/PAF.0b013e3181cfc658, PMid: 20110803 10.1097/PAF.0b013e3181cfc658

[12] Dennis MS, Lindesay J. Suicide in the elderly: the United Kingdom perspective. Int Psychogeriatr. 1995;7(2):263–274. http://dx.doi.org/10.1017/S104161029500202X 8829432 10.1017/s104161029500202x

[13] Meel B. Epidemiology of suicide by hanging in Transkei, South Africa. Am J Forensic Med Pathol. 2006;27(1):75–78. http://dx.doi.org/10.1097/01.paf.0000202738.28446.4a, PMid: 16501355 10.1097/01.paf.0000202738.28446.4a

[14] Meel BL. Suicide in HIV/AIDS in Transkei, South Africa. Anil Aggrawal's Internet Journal of Forensic Medicine and Toxicology. 2003;4(1):1–9.

[15] World Health Organization Report on a workshop on suicide prevention for countries in the African region. Geneva: Department of Mental Health, World Health Organization; 1999.

[16] Flisher AJ, Parry CD. Suicide in South Africa. An analysis of nationally registered mortality data for 1984-1986. Acta Psychiatr Scand. 1994;90(5):348–353. http://dx.doi.org/10.1111/j.1600-0447.1994.tb01605.x, PMid: 7872039 10.1111/j.1600-0447.1994.tb01605.x

[17] Sukhai A, Harris C, Moorad RG, et al. Suicide by self-immolation in Durban, South Africa: a five-year retrospective review. Am J Forensic Med Pathol. 2002;23(3):295–298. http://dx.doi.org/10.1097/00000433-200209000-00020, PMid: 12198362 10.1097/00000433-200209000-00020

[18] Roberts K, Wassenaar D, Canetto SS, et al. Homicide-suicide in Durban, South Africa. J Interpers Violence. 2010;25(5):877–899. http://dx.doi.org/10.1177/0886260509336964, PMid: 19617417 10.1177/0886260509336964

[19] Jena S, Mountany L, Muller A. A demographic study of homicide-suicide in the Pretoria region over a 5 year period. J Forensic Leg Med. 2009;16(5):261–265. http://dx.doi.org/10.1016/j.jflm.2008.12.009, PMid: 19481707 10.1016/j.jflm.2008.12.009

[20] Lester D. Personal violence (suicide and homicide) in South Africa. Acta Psychiatr Scand. 1989;79(3):235–237. http://dx.doi.org/10.1111/j.1600-0447.1989.tb10250.x, PMid: 2711848 10.1111/j.1600-0447.1989.tb10250.x

[21] Statistics South Africa South African population statistics In: Pretoria: Government Gazette; 2001.

[22] Donson H, editor. A profile of fatal injuries in South Africa Tygerburg: MRC/UNISA Crime, Violence and Injury Lead Programme; 2007 PMCid:

[23] Schlebusch L, Govender RD. Age, Gender and Suicidal Ideation Following Voluntary HIV Counseling and Testing. Int J Environ Res Public Health. 2012;9(2):521–530. 22470307 10.3390/ijerph9020521 PMC3315261

[24] Naidoo T, Kirk GM. Post-mortem analysis of all deaths. In: Faculty Research Day Durban: University of Natal; 2004.

[25] Matzopoulos R, Norman R, Bradshaw D. The burden of injury in South Africa: fatal injury trends and international comparisons. Tygerberg: Medical Research Council-University of South Africa, Crime, Violence and Injury Lead Programme; 2004.

[26] Flisher AJ, Liang H, Laubscher R, et al. Suicide trends in South Africa, 1968--90. Scand J Public Health. 2004;32(6):411–418. http://dx.doi.org/10.1080/14034940410029469, PMid: 15762025 10.1080/14034940410029469

[27] Mgaya E, Kazaura MR, Outwater A, et al. Suicide in the Dar es Salaam region, Tanzania, 2005. J Forensic Leg Med. 2008;15(3):172–176. http://dx.doi.org/10.1016/j.jflm.2007.06.002, PMid: 18313013 10.1016/j.jflm.2007.06.002

[28] Bertolote JM. Suicide in the world: an epidemiological overview 1959–2000. London: Martin Dunitz; 2001. PMid:

[29] Kõlves K. Child suicide, family environment, and economic crisis. Crisis. 2010;31(3):115–117. http://dx.doi.org/10.1027/0227-5910/a000040, PMid: 20573604 10.1027/0227-5910/a000040

[30] Masocco M, Pompili M, Vanacore N, et al. Completed suicide and marital status according to the Italian region of origin. Psychiatr Q. 2010;81(1):57–71. http://dx.doi.org/10.1007/s11126-009-9118-2, PMid: 20041348 10.1007/s11126-009-9118-2

[31] Bradshaw D, Masiteng K, Nannan N. Health status and determinants. Durban: Health Systems Trust; 2000.

[32] Schlebusch L. An overview of suicidal behaviour in Africa In: DM Ndetei, DM Szabo, editors. Contemporary psychiatry in Africa: a review of theory, practice and research. Nairobi, Kenya: Acrodile Publishing Limited, 2011; p. 375–396.

[33] Pillay AL, Wassenaar DR. Recent stressors and family satisfaction in suicidal adolescents in South Africa. J Adolesc. 1997;20(2):155–162. http://dx.doi.org/10.1006/jado.1996.0073, PMid: 9104651 10.1006/jado.1996.0073

[34] Noor Mahomed SB, Selmer CA, Bosch BA. Psychological profiles of children presenting with suicidal behaviour, with a specific focus on psychopathology In: L Schlebusch, BA Bosch, editors. Suicidal behaviour 4: Proceedings of the fourth Southern African conference on suicidology. Durban: University of Natal, 2000; p. 56–70.

[35] Schlebusch L. Suicidal behaviour In: A van Niekerk, S Suffla, M Seedat, editors. Crime, violence and injury in South Africa: enabling child safety. Pretoria, South Africa: Psychological Society of South Africa, 2012; p. 178–194.

[36] McMillan KA, Enns MW, Asmundson GJ, et al. The association between income and distress, mental disorders, and suicidal ideation and attempts: findings from the Collaborative Psychiatric Epidemiology Surveys. J Clin Psychiatry. 2010;71(9):1168–1175. http://dx.doi.org/10.4088/JCP.08m04986gry, PMid: 20441719 10.4088/JCP.08m04986gry

[37] Wassenaar DR, Pillay AL, Descoins S, et al Patterns of suicide in Pietermaritzburg 1982–1996: race, gender and seasonality In: L Schlebusch, BA Bosch, editors. Proceedings of the fourth Southern African conference on suicidology: University of Natal, 2000; p. 97–111.

[38] Vawda N. Suicidal behaviour among black South African children and adolescents In: S Malhotra, editor. Mental disorders in children and adolescents: need and strategies for intervention. Delhi: CBS Publishers and Distributors, 2005; p. 94–100.

[39] Meel BL. Determinants of suicide in the Transkei sub-region of South Africa. J Clin Forensic Med. 2003;10(2):71–76. http://dx.doi.org/10.1016/S1353-1131(03)00038-5 15275024 10.1016/S1353-1131(03)00038-5

[40] Burrows S, Laflamme L. Suicide mortality in South Africa: a city-level comparison across socio-demographic groups. Soc Psychiatry Psychiatr Epidemiol. 2006;41(2):108–114. http://dx.doi.org/10.1007/s00127-005-0004-4, PMid: 16362168 10.1007/s00127-005-0004-4

[41] Dzamalala CP, Milner DA, Liomba NG. Suicide in Blantyre, Malawi (2000–2003). J Clin Forensic Med. 2006;13(2):65–69. http://dx.doi.org/10.1016/j.jcfm.2005.08.006, PMid: 16271492 10.1016/j.jcfm.2005.08.006

